# The Health Impact Fund: making the case for engagement with pharmaceutical laboratories in Brazil, Russia, India, and China

**DOI:** 10.1186/s12992-021-00744-x

**Published:** 2021-09-06

**Authors:** Vivian Chia-Jou Lee, Jacqueline Yao, William Zhang

**Affiliations:** 1 Department of Pharmacology and Therapeutics; Faculty of Medicine and Health Sciences, McGill University, Montreal, Canada; 2 Department of Microbiology and Immunology; Faculty of Medicine and Health Sciences, McGill University, Montreal, Canada

**Keywords:** Pharmaceutical innovation, R&D, drug development, LMIC, BRIC, Health Impact Fund, Public-private-partnership, Health inequity, Social impact investing

## Abstract

Despite progress in global health, the general disease burden still disproportionately falls on low- and middle-income countries. The health needs of these countries’ populations are unmet because there is a shortage in drug research and development, as well as a lack of access to essential drugs. This health disparity is especially problematic for diseases associated with poverty, namely neglected tropical diseases and microbial infections. Currently, the pharmaceutical landscape focuses on innovations determined by profit margins and intellectual property protection. To expand drug accessibility and catalyze research and development for neglected diseases, a team of researchers proposed the Health Impact Fund as a potential solution. However, the fund is predominantly considering partnerships with pharmaceutical giants in high-income countries. This commentary explores the limitations and benefits in partnering with pharmaceutical companies based in Brazil, Russia, India, and China (BRIC), with the goal of expanding the Health Impact Fund’s vision to incorporate long-term, local partnerships. Identified limitations to a BRIC country partnership include lower levels of drug development expertise compared to their high-income pharmaceutical counterparts, and whether the Health Impact Fund and the participating stakeholders have the financial capability to assist in bringing a new drug to market. However, potential benefits include the creation of new incentives to fuel competitive local innovation, more equitable routes to drug discovery and development, and a product pipeline that could involve stakeholders in lower- and middle-income countries. Our commentary explores how partnership with pharmaceutical firms in BRIC countries might be advantageous for all: The Health Impact Fund, pharmaceutical companies in BRIC economies, and stakeholders in low- and middle- income countries.

## Introduction

### The Health Impact Fund as an incentive to catalyze R&D and expand drug accessibility

The Health Impact Fund (HIF) is a prospective pay-for-performance mechanism that encourages biopharmaceutical innovation by awarding payments commensurate with achieved health impact. The HIF is a project by the United States (US)-based not-for-profit organization Incentives for Global Health [1]. Through the HIF, the pharmaceutical industry’s incentives could be better aligned with social goals—namely, research and development (R&D) on neglected diseases that primarily affect lower- and middle-income countries (LMICs). According to *The Health Impact Fund: Making New Medicines Accessible for All*, the HIF aims to “give pharmaceutical innovators stable financial incentives’’ for developing new medicines that provide considerable health impacts. These drugs would then be sold at minimum prices that cover production and distribution costs[2]. Under this model, both newly registered drugs and repurposed compounds would be eligible for rewards paid by the HIF. Drugs would be rewarded based on their annual health impact, and be assessed by metrics similar to those used by the National Institute for Health and Clinical Excellence (NICE). While this assessment may be measured in Quality-Adjusted Life Years (QALYs), it is acknowledged that no global health impact assessment is perfectly accurate [2]. Additionally, the HIF would set a maximum reward per QALY and would allow at most 20 drug registrations at any time. In all, the HIF plans to give financial incentives to pharmaceutical companies for developing drugs that would otherwise be unprofitable in the HIF’s absence. The HIF seeks to be funded by federal governments, at 0.03 % of participating countries’ gross national income, ultimately amassing $6 billion USD per year in investments. More specifically, the HIF expects contributions to be made from all four World Bank income groups as it would contribute to realizing the internationally agreed Sustainable Development Goals and reduce dangers from invasive diseases that transcend national borders [1].

### Lack of access to essential drugs as a determinant of global health inequity

The end of 2020 marks only ten years left to accomplish the United Nations's Sustainable Development Goals (SDGs). SDG 3 presents a plan toward Good Health and Well-being, with a main tenet being universal health coverage and “access to safe, effective, quality and affordable essential medicines and vaccines for all” [3].

One potential obstacle to achieving SDG 3 dates back to 1994: the World Trade Organization (WTO) agreement on Trade-Related Aspects of Intellectual Property Rights (TRIPS). TRIPS enforces patent protection across WTO member nations, including Efavirenz (generic ARV drug), Plavix (antiplatelet medication) and many others on the WHO Model List of Essential Medicines [4]. To offer greater flexibility in the face of public health emergencies like the global AIDS crisis, a set of mechanisms allowing states to circumvent patent laws was created. The 2001 Doha Declaration on the TRIPS Agreement and Public Health is one such mechanism, but this may not be enough in the face of quotidian health inequalities [5]. This is because, despite the ability to issue compulsory licenses and allowing parallel importing under Article 31*bis*, the Declaration was received in a way that Members would only use the system in “situations of national emergency or other circumstances of extreme urgency” [6]. These issues are most relevant in low- and lower middle-income countries that face greater challenges in accessing medicine. In 2001, Least Developed Country Members (“deemed to have insufficient or no manufacturing capacities in the pharmaceutical sector”) within the WTO were given a waiver for TRIPS obligations related to intellectual property rights on pharmaceutical products and clinical data, enabling the purchase and production of generic medicines. While this waiver was extended to 2033 [7], the declaration does not address the shortage of drugs for neglected, endemic diseases. The proposed HIF strives to provide LMICs with high-impact medicines that would otherwise either sell at high, patent-protected prices, or not be developed at all. This is the connection between TRIPS and the proposed HIF mechanism: TRIPS limits the availability of low-cost, generic versions of existing drugs in LMICs, and the HIF has the potential to incentivize R&D toward new drugs that treat diseases for which the provision of generic counterparts is being limited. The HIF plans to financially reward drugs based on impact—an opportunity to provide pharmaceutical companies an (otherwise predominantly absent) incentive to develop drugs for diseases primarily affecting LMICs—thereby a way to delink drug prices from R&D costs.

### New incentives are needed for research and development of essential medicines

The second major barrier in providing essential medicines for all is the stagnation of R&D investment in the health needs of LMICs. Three-quarters of the world’s population lives in LMICs, but these countries’ collective R&D spendings account for less than 10 % of the global pharmaceutical expenditure [8]. Inhabitants of LMICs face higher disease burdens—both communicable and non-communicable—but the majority of pharmaceutical companies focus on developing drugs for non-communicable diseases within high-income countries. Big pharmaceutical companies, protected by patent monopolies and market exclusivities, can generate revenue to fund future R&D and recoup marketing costs by charging high prices [9]. This leaves little room and incentive for R&D on neglected tropical diseases, antibiotics, and drugs that help LMICs; R&D in these areas may be considered inefficient from a revenue standpoint. Therefore, issues surrounding accessibility and affordability of essential medicines still remain [10].

### Existing private-public partnership initiatives and delayed implementation of HIF

Alternatives to traditional, profit-driven drug development models have emerged in the early 2000s to address the shortage of drugs for neglected, communicable diseases. These include the TB Alliance, the International AIDS Vaccine Initiative, Medicines for Malaria Venture, and Drugs for Neglected Diseases Initiative (DNDi). The HIF differs from these aforementioned initiatives in that the HIF seeks to support potentially high impact drugs indiscriminate of disease conditions (communicable or non-communicable), and that it introduces a pay-for-performance mechanism to motivate competition. In theory, this creates incentives for registrants to create highly efficacious drugs targeting common diseases. However, it has been 13 years since the HIF was first proposed, and the fund is still in a nascent stage of development. This delay may be due to the absence of previous, large-scale pilot projects to demonstrate its feasibility and garner interest from potential private partners, which include investors and pharmaceutical companies. Additionally, the HIF has not acquired its proposed annual $6 billion in budget [2].

The HIF is presently gauging market interest as well as its own scalability. The HIF’s most recent development is a pilot proposal that aims to sponsor R&D projects for up to three years, though the $60-$200 million USD necessary to fund this does not yet seem to have been raised [11]. In order to grow its internal operations and gain buy-in from government funders, the HIF is looking to partner with organizations such as UNITAID and DNDi, or possibly an international drug-development-supporting organization such as the Helmholtz Research Institute.

## Problem Statement

The twentieth century saw the proliferation of healthcare technologies, as well as market dominance by large pharmaceutical companies. As a result, the HIF Pilot Project is interested in negotiating partnerships only with large, multinational pharmaceutical companies located in high-income countries. In a consultation held with HIF, an HIF executive stated that big pharma companies have more capacity to engage with proposals such as the Health Impact Fund. However, motives—financial or otherwise—for big corporations to associate with the HIF are not apparent. The HIF Pilot Project’s financial reward pool of $60–$200 million USD [11], once divided amongst multiple registrants, is likely a small fraction of the annual profit generated by multinational companies.

Alternatively, a solution for the HIF to generate buy-in could be to enlist pharmaceutical manufacturers within BRIC countries, as opposed to large pharmaceutical companies. Coined by economist Jim O’Neill, BRIC, an acronym for Brazil, Russia, India, and China, refers to economies that are at an advanced stage of development [12]. However, these countries face disease burdens that overlap with those from other LMICs. For example, over one third of India’s disability-adjusted life years are caused by communicable, maternal, perinatal, and nutritional diseases [13]. Neonatal and maternal illnesses have decreased substantially in Russia between 1980 and 2016, but communicable diseases such as HIV/AIDS and tuberculosis are still a public health concern [14]. While China and Brazil have seen decreases in diseases of poverty, both countries continue to face the dual threat of non-communicable disease and communicable disease burden [15, 16]. Thus, because BRIC countries have a population still facing diseases associated with low-income populations, they may be incentivized to partner with the HIF and develop drugs that address health concerns suitable for LMIC markets.

Partnering with pharmaceutical companies in BRIC countries could also create an opportunity for the HIF to make a global health impact for those living in LMICs. The lower gross profit and net profit potential of BRIC-based pharmaceutical manufacturers incentivize these firms to register their products with the HIF. Moreover, local pharmaceutical firms likely have local distribution advantages that facilitate vulnerable populations’ access to quality medicines, reduce dependency on international aid, and contribute to sustainable industrial development.

The aim of this commentary is to explore how partnering with pharmaceutical companies situated in BRIC countries can help the HIF establish itself and realize its goals. A literature search was conducted on PubMed and Google Scholar, with keywords such as: drug financing, neglected diseases, pharmaceutical research + BRIC, Health + Impact + Fund. The inclusion criteria for the chosen sources were: to be between the years 2000–2020, peer-reviewed, and from industries and/or governmental reports. Additionally, consultations with experts in the non-for-profit and the private equity sectors were made. These experts have consented to having their insights included in this commentary.

## Limitations of Working with Pharmaceutical Companies in BRIC Countries

Drug R&D is a capital-intensive process that is supported by government funding during early-stage research [17]. Therefore, to partner with pharmaceutical companies in BRIC countries, the HIF and participating governments need to determine whether the HIF model has sufficient financial strength to bring a new drug to market. The HIF’s financial prospects aside, partnering with BRIC-based pharmaceutical companies may present limitations. In the following subsections, we explore the lack of R&D expertise in pharmaceutical companies based in BRIC countries.

### Lack of R&D precedent in domestic industries

Although there have been advances in R&D within BRIC-based pharmaceutical companies, most of these companies continue to focus on producing generic medicines over novel therapeutics.

Despite having the world’s second largest pharmaceutical industry, China (a BRIC country) ranked below many high-income countries such as the United States, South Korea, Israel, and Japan for biomedical competitiveness in a 2017 global survey [18]. The survey captures 31 of the largest and most active pharmaceutical markets worldwide and provides an in-depth view of both emerging and mature pharmaceutical markets. It measures the sophistication of the biopharmaceutical systems, historical R&D and manufacturing capabilities. More specifically, biopharmaceutical competitiveness in China is hindered by gaps in quality control, a need for more public-private collaboration, and long trial approval timelines. Nonetheless, China’s capacity for clinical research is relatively developed [18].

Brazil has the largest pharmaceutical industry in Latin America, but its innovation competitiveness is ranked lower than average [18]. Private pharmaceutical companies often do not have the resources or facilities to develop new drug candidates, and much of their budget is spent on imported intermediate products to complete drug production [18]. Furthermore, R&D activities in Brazil are mostly conducted by educational institutions, maintaining little partnership with private companies [19]. Although clinical research capacity among hospitals are fairly developed, there seems to be a lack of long-term national policy to strengthen R&D [18].

Russia has seen an expansion in scientific capability resulting from increases in training and education [18]. However, even as the BRIC country with the highest GDP per capita, Russia’s pharmaceutical industry is stagnant from a lack of R&D investment and from high levels of equipment depreciation [20]. While good manufacturing practice (GMP) compliance has improved, industry executives may view Russia’s regulatory capacities as rudimentary[18].

Pharmaceutical companies in India are promising registrants for the HIF. India is the largest provider of generic pharmaceuticals and has a growing capacity of high-skilled researchers. Additionally, the capacity for drug review is adequate (albeit inconsistent) across regions [18]. But, weak technology infrastructure in India inhibits technology transfer, and intellectual property protection continues to be perceived as inadequate [18].

The race to develop COVID-19 vaccines has demonstrated the potential for newcomers to excel in pharmaceutical innovations. As of April 2021, five of the twelve unique COVID-19 vaccines approved in at least one country were developed by Chinese firms (Sinopharm, Sinovac, CanSino, Anhui Zhifei Longcom) [21]. Also, Russia and India have each developed one approved vaccine—Sputnik V and Covaxin, respectively. Therefore, despite BRIC countries’ perceived lack of R&D capability, the pharmaceutical innovation landscape may change in the coming decades.

## Benefits to Partnering with BRIC LMIC-Based Pharmaceutical Companies

Although large pharmaceutical companies have investment capabilities and a greater ability to absorb losses, the HIF’s pay-for-performance reward system may not incentivize these pharmaceutical giants into partnership.

With the HIF’s ideal annual budget of $6 billion USD and a plan to allocate $600 million USD per year towards administration and assessment [2], pharmaceutical companies could only receive an average of $275 million USD per year if the maximum of 20 HIF-registered drugs in a given period is reached. Pharmaceutical giants like Johnson & Johnson spent $12 billion USD in one year on research and development—$275 million USD is only 2.26 % of their 2020 expenditure [22]. Given that these estimates are made with the HIF’s best case funding scenario of $6 billion USD, the HIF Pilot Project ($60–$200 million USD) means even less financial reward for big pharma. Additionally, an asset management fund CEO stated during a consultation that $6 billion USD would not only be very difficult to obtain, but would also be financially insufficient for large pharmaceutical companies as a reward.

Hence, when exploring potential pharmaceutical registrants, we recommend that the HIF consider partnerships with BRIC-based pharmaceutical companies. The reasons can be separated into three categories:

1. Benefits for the HIF;
2. Benefits for the BRIC-Based Pharmaceutical Companies;
3. Benefits for the Affected Population.

### Benefits for the HIF

If the HIF seeks partnership with pharmaceutical companies based in BRIC countries, it can increase buy-in from BRIC governments, build sustainable and equitable partnerships within the local market-of-interest, and fuel the competition necessary for their pay-for-performance model.

By approaching BRIC-based pharmaceutical companies, the HIF motivates BRIC governments to contribute the necessary 0.03 % GNI investment [2]. The support that the HIF could provide for BRIC-based pharmaceutical companies allows BRIC and other LMICs to be less dependent on external markets, build the momentum necessary for local drug production, and sustain the 0.03 % GNI investment over a long duration—ultimately ensuring the HIF’s own survival.

Through direct partnership with BRIC countries, the HIF can help partnering countries meet at least four out of the seventeen Sustainable Development Goals (SDGs). If implemented successfully, the HIF can support BRIC in meeting: SDG 3 (Good Health and Wellbeing), SDG 9 (Industry, Innovation and Infrastructure), SDG 12 (Responsible Consumption and Production), and SDG 17 (Partnerships for the Goals).

Lastly, if the HIF partners with BRIC-based pharmaceutical companies, it helps foster the competition necessary for its proposed pay-for-performance model. Since the HIF’s current reward system is suitable for the needs of medium-sized pharmaceutical companies, a direct partnership can build competition amongst other medium-sized registrants, thus supporting the HIF’s goal to promote the development of novel therapeutics.

### Benefits for BRIC Pharmaceutical Companies

Since some pharmaceutical companies in BRIC are small and medium enterprises, they might lack the funds for advertisement, human resources, and strong relationships with regulatory entities [23]. To address these issues, BRIC-based pharmaceutical companies can reap the benefits of a partnership with the HIF by receiving public credibility, attracting high-end investigators, and leveraging the HIF’s partnership with local governments. These benefits are suited to incentivize BRIC-based pharma because large companies in high-income countries already have the resources to finance and capture stakeholder support for R&D.

The pharmaceutical sector spends a copious amount on direct-to-consumer advertising [24]. By registering a drug with the HIF, pharmaceutical firms in BRIC countries demonstrate through advertisements that are publically credibile in terms of good social responsibility and can gain public spotlight in doing so. In this vein, media coverage of an HIF-registered product may act as advertisement for drugs, medical devices, and diagnostics in the pharmaceutical company’s profile. In addition to gaining patients’ trust, a partnership with the HIF can attract investors that may otherwise be hesitant about a firm’s capabilities to develop or enter the market of novel therapeutics.

A partnership with the HIF could potentially help BRIC-based pharmaceutical firms to attract and retain research talent via prosperity in organisational performance and employee welfare. By aligning itself with the HIF, a firm could bolster employee perception of internal corporate social responsibility practices and demonstrate a mission that is not purely profit-driven. By aligning their firm’s vision with the HIF’s mandate, pharmaceutical companies could accrue benefits derived from the influencing mechanism of employee intrapreneurial behaviour [25]. Under an HIF partnership, biomedical scientists can support social responsibilities, capitalize on their skill-sets to help marginalized populations, and work on more high-impact projects.

If the HIF can secure direct partnerships with local BRIC governments, pharmaceutical companies based in these countries can then leverage governmental support to bypass regulatory hurdles, earn financial subsidy, and lower risks. Regulatory entities in BRIC economies can re-evaluate their systems and allow for fast-track designations, similar to those made by the US Food and Drug Administration to expedite drug development and review. In addition, innovative public-private-partnership strategies can result in both government grants for the initial R&D investment and an altered risk-adjusted rate of return. A venture capital fund CEO also suggested using methods such as “first-in, last-out”, by which governments can support the pharmaceutical industry in their upfront costs of R&D. The government can invest a fraction of the entire cost of drug development while the pharmaceutical company gathers the remaining sum from private investors. Once the product goes to market, private investors can gain a given rate-of-return, along with their original investment. This “first-in, last-out” schematic is attractive for private investors because they only incur a portion of the risk involved in a large investment.

### Benefits for BRIC Governments

The HIF–BRIC pharmaceutical partnership could benefit the local governments’ drug regulation system. If a drug originates from the USA, Europe, Japan, or elsewhere in the world, BRIC countries often face a lag time for domestic distribution of that drug. By conducting new drug R&D directly under the BRIC government’s own jurisdictions, the local population could receive immediate treatment and shorten wait-times caused by regulatory and patent approvals. Although the regulatory systems in BRIC countries may currently be underdeveloped in comparison to those in high income countries, a partnership with HIF could incentivize local governments to strengthen their regulatory systems and streamline their own post-market pharmacovigilance operations [26]. According to a paper published in Perspectives in Clinical Research, reduction of drug distribution lags in emerging markets is possible if key regulatory barriers such as “Western Approval” could be circumvented by the green light for production being given directly by BRIC governments [26]. Based on how pharmaceutical companies in BRIC economies have produced safe and efficacious COVID-19 vaccines, there is greater potential that BRIC firms can deliver treatments for LMICs [21].

By running R&D in countries-of-interest, the HIF can produce suitable drugs, prioritize patient needs, shorten the time lag between innovation and distribution, and strengthen pharmacovigilance in favor of the therapeutic users.

## Conclusions

As the HIF prepares to launch its pilot project, the HIF must attract eligible pharmaceutical firms that can submit projects that improve health impact. Currently, the HIF is depending solely on large pharmaceutical giants for potential buy-in. In hopes of broadening the HIF’s scope in its search for suitable registrants, we explored the limitations and benefits in partnering with pharmaceutical companies based in BRIC countries. Although the HIF may encounter limitations such as lack of R&D precedent, the HIF also has the potential to strengthen pharmacovigilance and ignite vigor in local R&D by prompting local governments to invest.

Although the HIF is focused on multinational corporations to submit drug candidates, the $60–$200 million USD reward pool is a small sum compared to the financial revenues of big pharmaceutical companies. Meanwhile, pharmaceutical companies based in BRIC economies may find the HIF’s reward system more appealing, and pose as more realistic targets for buy-in. These outlined partnerships can serve the HIF’s proposed R&D mechanism by encouraging BRIC governments to fund and fuel the competition necessary for a pay-for-performance model. Hence, registering novel drugs produced by BRIC-based pharmaceutical companies will ostensibly support the HIF’s goal of increasing R&D and access to medicines. If the HIF considers these recommendations and seeks projects with BRIC-based pharmaceutical companies, it could lay a much-needed foundation for equitable and sustainable partnerships.

## Data Availability

Not applicable.
